# Simulation-based assessment of model selection criteria during the application of benchmark dose method to quantal response data

**DOI:** 10.1186/s12976-020-00131-w

**Published:** 2020-08-05

**Authors:** Keita Yoshii, Hiroshi Nishiura, Kaoru Inoue, Takayuki Yamaguchi, Akihiko Hirose

**Affiliations:** 1 Graduate School of Medicine, Hokkaido University, Kita 15 Jo Nishi 7 Chome, Kita-ku, Sapporo-shi, Hokkaido 060-8638 Japan; 2 CREST, Japan Science and Technology Agency, Honcho 4-1-8, Kawaguchi, Saitama, 332-0012 Japan; 3 Division of Risk Assessment, National Institute of Health Sciences, Kawasaki, Japan; 4 The Center for Data Science Education and Research, Shiga University, 1-1-1 Banba, Hikone-city, Shiga 522-8522 Japan

**Keywords:** Risk assessment, Dose-response curve, Toxicology, Simulation, Model averaging, Benchmark dose

## Abstract

**Background:**

To employ the benchmark dose (BMD) method in toxicological risk assessment, it is critical to understand how the BMD lower bound for reference dose calculation is selected following statistical fitting procedures of multiple mathematical models. The purpose of this study was to compare the performances of various combinations of model exclusion and selection criteria for quantal response data.

**Methods:**

Simulation-based evaluation of model exclusion and selection processes was conducted by comparing validity, reliability, and other model performance parameters. Three different empirical datasets for different chemical substances were analyzed for the assessment, each having different characteristics of the dose-response pattern (i.e. datasets with rich information in high or low response rates, or approximately linear dose-response patterns).

**Results:**

The best performing criteria of model exclusion and selection were different across the different datasets. Model averaging over the three models with the lowest three AIC (Akaike information criteria) values (MA-3) did not produce the worst performance, and MA-3 without model exclusion produced the best results among the model averaging. Model exclusion including the use of the Kolmogorov-Smirnov test in advance of model selection did not necessarily improve the validity and reliability of the models.

**Conclusions:**

If a uniform methodological suggestion for the guideline is required to choose the best performing model for exclusion and selection, our results indicate that using MA-3 is the recommended option whenever applicable.

## Background

**Fig. 1:**
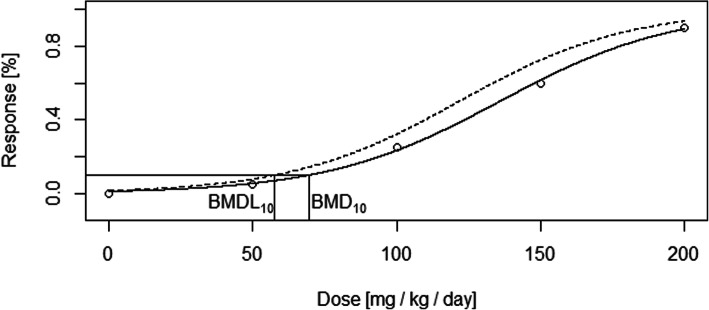
Example of benchmark dose (BMD), BMDL in Dose-response curve. A dose-response curve illustrating relationship between BMD_10_ and BMDL_10._ Dots: fraction of animals affected in each dose group; Solid curve: Fitted model; BMD_10_: BMD corresponding to 10% extra risk on this curve based on fitted model; Dashed line: the estimated lower bound on doses for a range of BMRs; BMDL_10_: The lower bound on BMD_10_ based on the dashed curve

To determine the reference dose of chemical substances, including food additives and agricultural chemicals, that cause the presence or absence of a harmful event (i.e. dichotomous outcome) so that the acceptable daily intake can be specified, a number of scientific approaches using dose-response experimental data have been used. A popular toxicological method uses the responses to low dose exposures to confirm the absence of an outcome event. The highest dose that does not cause an event is referred to as the no observable adverse effect level (NOAEL), below which no outcome is expected. Multiplying this with a specified uncertainty factor that addresses uncertainties, including the biological species barrier between experimental animals and humans [1], the point of departure has been determined in practice. However, the determination of NOAEL depends on low dose data alone; so, if the number of experimental animals per dose is limited, this imposes a serious statistical limitation that involves non-negligible sampling errors [2, 3]. An alternative method, the benchmark dose (BMD) method, was initially formalized by Crump [4]. The BMD method determines the threshold dose by fitting various statistical models to the dose-response curve, which addresses the problems surrounding the use of NOAEL because it can account for the response data across different doses and can help in objectively calculating the point of departure. The BMD method can potentially be extremely useful in many scientific disciplines [5, 6]. The benchmark dose lower bound (BMDL), which is the lower (one-sided) limit of the 95% confidence interval of BMD, can yield a point of departure that is comparable to that based on NOAEL (Fig. 1) [7, 8].

To employ the BMD method, it is critical to select the best performing BMDL by following the statistical fitting procedures of multiple mathematical models. Parameterized models only characterize reality, so multiple models (usually nine or more) are commonly fitted to the same experimental dataset. As a result, many BMDL values can act as the candidate of preferred reference dose. However, the reference dose should be the best performing BMDL and it must be selected, for example, as the one that gives the best fitting results [9]. There are two additional issues in selecting or determining the BMDL. First, the BMD method uses a specified percentile point (e.g. 10% of the benchmark response, abbreviated as BMD_10_) as the threshold for the reference value, but the 10% percentile point is never strictly objective [10–12]. This is similar to using a *p*-value of 5% in many hypothesis tests or other arbitrarily chosen threshold values. Second, some fitted models (e.g. the Weibull model) yield different parameter estimates when restrictions to the range of parameters are imposed in advance of the inference procedure [9]. Quantitative guides for such restrictions can be complicated for non-expert users.

Although several technical problems exist, we believe that the biggest obstacle to the wide application of the BMD method in various governmental settings is the lack of uniform guidelines that specify the steps required to scrutinize fitting results and identify a single BMDL value for determining the acceptable daily intake. Objective guidelines are required to determine which candidate models should be included or excluded in the final evaluation report. Model exclusion has been attempted by goodness-of-fit testing and by measuring arbitrarily defined marker of fit, e.g., the ratio of BMD to BMDL [13–15]. Nevertheless, model exclusion has not been consistently practiced (i.e. sometimes not conducted) and the criteria of exclusion have not been verified and/or harmonized across different studies. Further, more model selection methods have been discussed and developed [16, 17] than exclusion methods. A conservative approach is to use the modeling result that yields the lowest BMDL among all the fitted models [10, 18], which was recommended by the European Food Safety Authority in 2009 [19]. However, the model with the lowest BMDL might be the model with the broadest uncertainty; i.e. a wide confidence interval caused by a bad fit (e.g. even a fitted model can be rejected by Pearson’s chi-squared test [10]). The AIC (Akaike information criteria) [16, 20] or BIC (Bayesian information criteria) [16] could be used as alternative ways of measuring goodness-of-fit and selecting the model that gives the lowest value (i.e. the best fit model). However, having the lowest AIC does not guarantee that the goodness-of-fit of the model around the low dose response will be successful and valid to yield an appropriate BMDL [10, 16]. Model averaging has been proposed as a possible solution [21–23] that may partly resolve the uncertainties associated with the use of mathematical models to explain the dose-response data. An updated document in 2017 from the European Food Safety Authority [24] recommends the selection of multiple models with close AIC values (within ±2) and averaging the results from all the selected models. Model averaging has been proposed in various risk assessment settings using dose-response data [25–27], but a standard application method of model averaging has yet to be decided, including the use of badly fitted model for averaging (e.g. model averaging over all converged models or averaging over well-fitted models only).

While all issues surrounding the use of the BMD method for quantal response data cannot be fully and immediately resolved, a simulation-based evaluation might help to identify a possible well-performing pathway of model exclusion and selection. To support the formulation of technical guidelines for risk assessment practices for food safety in Japan, we conducted a simulation study to compare the performance of each and various combinations of model exclusion and selection criteria, as applied to three qualitatively different types of quantal response datasets.

## Methods

### Quantal response data

For the simulation-based assessment, we selected three datasets that are qualitatively different; i.e., (i) a dataset with frequent testing at doses with high response rates, (ii) a dataset with frequent testing at doses with low response rates, and (iii) a dataset with doses involving both high and low response rates. Specifically, the data were retrieved from animal experiments with (i) 1-aminoanthraquinone with an outcome of eosinophilic droplet in proximal tubular epithelium in kidney in male rats [28], (ii) 2-ethylhexyl vinyl ether with an outcome of centrilobular hypertrophy in liver stem cells in male rats [29], and (iii) acrylamide with an outcome of axon degeneration in peripheral nerve in male rats [30] as datasets (i), (ii), and (iii), respectively. In this study, we were not concerned with the biological properties of the experimental results or interpretations for toxicological assessment, rather we manually selected these datasets purely on the basis of the qualitative patterns of the observed dose-response curves. The sample size for each determined dose was *n* = 13, 6, and 48 and the original study examined responses at 4, 4, and 5 different doses (thus involving a total of 52, 24 and 240 exposed animals in datasets (i), (ii), and (iii), respectively).

Using the total of nine different distributions that consist of 2–4 unknown parameters, the BMD method was employed to analyze the datasets. For each dataset, we first identified the best-fit model by selecting the model with the lowest AIC value, without imposing any parameter restrictions and without excluding any models in advance of model selection. The nine statistical models used in this study were:

- Logistic model: $$ \frac{1}{1+\exp \left(-a- bx\right)} $$,
- Probit model: *Φ*(*a* + *bx*),
- Log-logistic model: $g+\frac{1-g}{1+\exp \left(-b-c\log (x)\right)}$,
- Log-probit model: *g* + (1 − *g*)*Φ*(*b* + *c* log(*x*)),
- Gamma model: $$ g+\left(1-g\right)\frac{1}{\varGamma (a)}{\int}_0^{bx}\left({t}^{a-1}\exp \left(-t\right)\right) dt $$,
- Weibull model: *g* + (1 − *g*)(1 − exp(−*ax*^*b*^)),
- Multistage (quadratic) model: *g* + (1 − *g*) exp(−*ax* − *bx*^2^),
- Multistage (cubic) model: *g* + (1 − *g*) exp(−*ax* − *bx*^2^ − *cx*^3^),
- Quantal-linear model: *g* + (1 − *g*) exp(−*ax*),

where *a*, *b*, and *c* represent unknown parameters, *g* is also an unknown parameter but it is used to represent the baseline response value for 0 ≤ *g* < 1, *x* is the dose, *Φ*(*x*) is the cumulative distribution function of the standard normal distribution at dose *x*, and *Γ*(*x*) is the gamma function at dose *x*. During the simulations, we regarded the identified best model for each chemical substance as the “reference model”. Such a true model is accompanied by the known lower bound of the benchmark dose with response level at 10% (i.e. unbiased BMDL_10_) as derived from the maximum likelihood estimates of the parameters.

The statistical estimation was conducted using the maximum likelihood method, and the likelihood function was defined under the assumption that the quantal response data at a given dose follows a binomial distribution. Computation of the 95% confidence interval (CI), including BMDL and BMD upper bound (BMDU) (i.e. one-sided upper 95% CI of BMD), relied on the bootstrapping method. Specifically, case resampling was performed using the Monte Carlo algorithm. We did not use the profile likelihood method to avoid a too conservative (underestimated) CI. We also did not use the parametric bootstrapping, because the sample sizes in the original datasets were small, and the use of multivariate normal distribution was not fully supported.

### Simulation-based evaluation

We performed a simulation-based assessment of model performance using the three “reference models” with three different dose response curves. Briefly, our analysis goes by: (i) identification of a reference model for each dataset by AIC (Akaike Information Criteria), (ii) generation of a total of 1000 simulated datasets (each dataset includes fittings by 9 individual model) from the reference model, (iii) application of model exclusion criteria if available, (iv) application of one of the model selection criteria including methods using model averaging, and select or calculate one of the representative BMDL value from each dataset, and (v) BMDL values were evaluated in two aspects, the validity and the reliability.

**Fig. 2:**
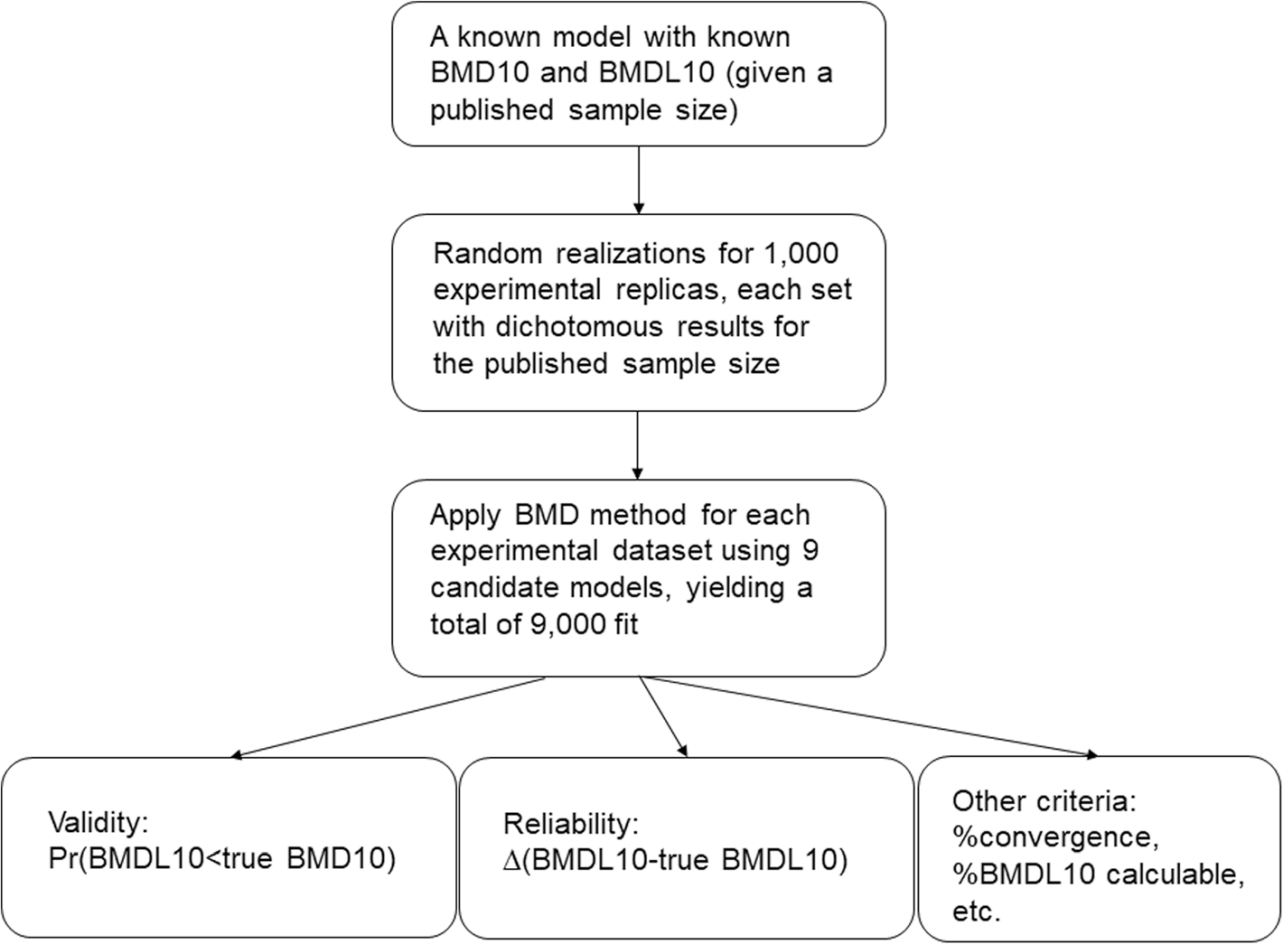
Simulation-based assessment of model selection criteria using the benchmark dose (BMD) method. We used three different datasets, 1-aminoanthraquinone, 2-ethylhexyl vinyl ether, and acrylamide. For each dataset, unbiased estimates were obtained for the benchmark dose that was 10% of the benchmark response (BMD_10_) and the lower bound of the benchmark dose with response level at 10% (unbiased BMDL_10_). Random realizations for generating 1000 replicas of the experimental data were conducted and applied the BMD method with nine models for each replica, which generated 9000 different model fits that were evaluated

Because of the statistical estimation that we performed, we considered that we knew the unbiased BMD_10_ and unbiased BMDL_10_ values that should be recovered by the BMD method using the simulated datasets. Specifically, we randomly generated a total of 1000 simulated datasets from the reference model (Fig. 2). The response outcome data were randomly generated from a binomial distribution for each examined exposure dose for the number of samples that were originally allocated for the given dose (i.e. *n* = 13, 6, and 48 dichotomous responses in each observation dose for the substances in datasets (i), (ii), and (iii)). For each replicated dataset, we fitted a total of nine standard distributions of the BMD method and examined whether an appropriate BMDL_10_ value could be recovered. To recover BMDL_10_ values, we imposed different combinations of model exclusion and selection criteria, which allowed us to assess which criteria would likely produce a valid and reliable estimate. The candidate of selection method includes the model averaging. To determine if the simulated criteria was valid and reliable, we evaluated the performance as follows:

(i) Validity.

The simulated BMDL_10_ value must be the dose lower than the unbiased BMD_10_ because the statistical role of BMDL is to act as the one-sided 95% lower bound of BMD. Out of the total of 1000 simulations for each chemical substance, the validity was measured as

$$ \frac{1}{1000}{\sum}_{i=1}^{1000}{I}_{ij}\times 100\ \left(\%\right), $$

$$ {I}_{ij}=\left\{\begin{array}{c}1\ \mathrm{if}\ {l}_{ij}<B\\ {}0\kern0.28em \mathrm{if}\ {l}_{ij}\ge B\end{array},\right. $$

where *l*_*ij*_ is the BMDL_10_ value based on the *i*-th simulated data and determined using model exclusion and selection criteria *j*, and *B* is the unbiased BMD_10_ value.

(ii) Reliability.

For the criteria to be reliable, similar results must be reproduced by repeating the same experiments. That is, the simulated BMDL_10_ value must be close to the unbiased BMDL_10_ value, and criteria that yield a “distant” BMDL_10_ value from the unbiased one would be regarded as a bad combination. Reliability was measured quantitatively as the relative distance from the unbiased BMDL_10_ as

$$ \frac{1}{1000}{\sum}_{i=1}^{1000}\frac{{\left({l}_{ij}-L\right)}^2}{L}, $$

where *L* is the unbiased BMDL_10_ value.

In addition to validity and reliability, we also assessed the calculability of the BMDL value. That is, the proportion of simulated datasets that yielded convergence and thus the BMDL value out of the total simulated datasets (1000) was assessed. Moreover, to avoid the impact of substantial exclusions before model selection on the calculability assessment, we also calculated the same proportion out of the total simulated datasets that survived the model exclusion process. Lastly, as a potential pitfall of simulation-based studies, it is important to remember that the original true model is likely to be recovered more often than other models, especially if a higher number of samples is tested for each dose. To avoid overoptimistic interpretation of the simulated results, we calculated the proportion of the same statistical model, out of the nine candidate models, that was recovered to be identical to the original (i.e. reference) model out of 1000 simulated datasets. If the selected statistical model was the same as the reference model, it is possible that the corresponding result may have been recovered due to the computational nature of the simulation study (e.g. random simulations using the Weibull model may lead to the choice of the Weibull model in each simulation run).

### Model exclusion and selection criteria

We considered a total of four possible model exclusion criteria and six possible model selection criteria. Avoiding excessive combinations of the two (i.e. multiple exclusion criteria plus model averaging over preferred models only), we tested and compared a total of 18 possible combinations.

The four model exclusion criteria were (i) no exclusion, (ii) implementing goodness-of-fit testing using the Kolmogorov-Smirnov test (KS test) to avoid models with *p* < 0.10, (iii) KS test to exclude models with p < 0.10 and also exclusion of models with the BMD/BMDL ratios > 10, and (iv) KS test to exclude models with p < 0.10 and also exclusion of models with BMDU/BMDL ratios > 10 [31]. We used the KS test rather than Pearson’s chi-squared or Fisher’s exact test because the experimental sample sizes were very small [31–33]. BMD/BMDL and BMDU/BMDL ratios > 10 were excluded because models with ratios that exceed 10 have been regarded as precise enough to yield a proper confidence limit [34–36]. Only models that survived these exclusion procedures were used in the model selection process.

Among the six model selection criteria, three were single selection criteria and three were model averaging methods. For the single selection criteria: (i) select the model with the lowest BMDL value to be conservative as part of risk assessment practice (Lowest BMDL) [10, 18]; (ii) select the model with the lowest BMD value, not necessarily relying on the lower uncertainty bound (Lowest BMD) [14]; or (iii) select the model with the lowest AIC value as the best fit model (Lowest AIC) [14]. We also computed model averaging results, not by taking the average BMDL value, but by averaging all or part of the fitted models for each resampled data. Model averaging takes into account the model uncertainty by integrating results from all or selected models [26, 37–40]. We considered three different patterns of model averaging: (i) model averaging over all nine models (MA-all) [25]; (ii) model averaging over three models with the lowest three AIC values (MA-3) [25]; and (iii) model averaging over all models that yielded AIC values within 3 of the lowest AIC value (MA-AIC). Let *π*_*i*_(*d*) the dose-response curve of *i*-th model and *d* the given dose, MA-all was calculated as ${\pi}_{\mathrm{MAall}}(d)={\sum}_{i=1}^9{w}_i{\pi}_i(d)$ where ${w}_k=\frac{\exp \left(-{I}_k/2\right)}{\sum_{i=1}^9\exp \left(-{I}_i/2\right)}$ and *I*_*k*_ is the AIC value of model *k*. MA-3 was calculated using the same formula with normalization over the three best-fit models as judged by AIC. Similarly, MA-AIC was computed using the arithmetic average of models (i.e. averaging without weight function) and adhering to rules of thumb [17], averaging all models with AIC within 3 of the lowest AIC of the best-fit model. The weight function was not used for MA-AIC because, in this instance, models with similar AIC values are regarded as equally well fitted models. MA-3 and MA-AIC are intended to conduct averaging over well fitted models compared with MA-all, so we did not examine a combination of model exclusion with MA-3 or MA-AIC to avoid similar removal of bad-fit models multiple times.

## Results

**Fig. 3:**
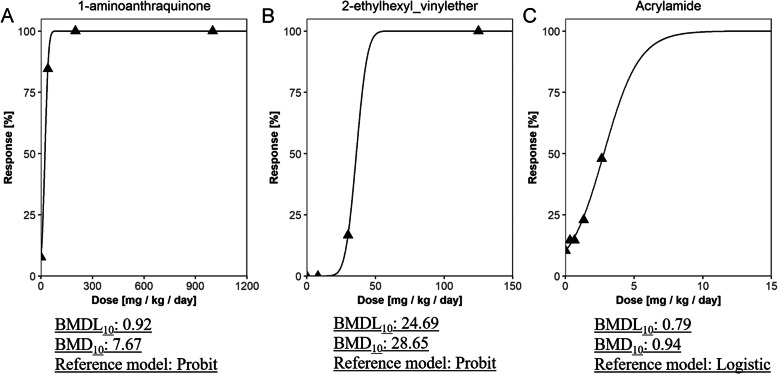
Observed and predicted dose-response relationships for the 1-aminoanthraquinone, 2-ethylhexyl vinyl ether, and acrylamide datasets. **a** 1-aminoanthraquinone with a substantial weight applied for doses with high response rates (*n* = 13 per observed dose). **b** 2-ethylhexyl vinyl ether with a weight applied for doses with low response rates (*n* = 6 per dose). **c** Acrylamide with an approximately linear dose-response relationship (*n* = 48 per dose). Original outcomes were eosinophilic droplet in renal proximal tubular epithelium in male rats for 1-aminoanthraquinone, centrilobular hypertrophy in liver stem cells in male rats for 2-ethylhexylvinyl ether, and axon degeneration in peripheral nerve in male rats for acrylamide, which we disregard in this study. The best fit models selected using only the Akaike information criterion were the Probit model for 1-aminoanthraquinone and 2-ethylhexyl vinyl ether, and the Logistic model for acrylamide. Unbiased BMDL_10_ and BMD_10_ were estimated as 0.9 and 7.7 respectively, for 1-aminoanthraquinone, 24.7 and 28.7, respectively, for 2-ethylhexylvinyl ether, and 0.8 and 0.9, respectively, for acrylamide

The validity and reliability of the simulation results for the 1-aminoanthraquinone dataset, which contained frequent testing at doses with high response rates, are listed in Table 1. BMDL_10_ was 0.92 and BMD_10_ was 7.67 under the selection of Probit model as the reference model with the lowest AIC value (Fig. 3), and resampling-based simulations were performed. The lowest BMDL or lowest BMD yielded the best validity results, except when the exclusion using the KS test and BMDU/BMDL ratio was applied in advance of model selection. The lowest BMD following model exclusion using both the KS test and BMD/BMDL ratio yielded the best reliability results. The lowest AIC was among the worst criteria, although about 1/3 of simulation results selected by the lowest AIC were produced by the Probit model, i.e. the reference model. Model averaging results yielded intermediate ranks among all model exclusion and selection criteria, and MA-3 produced the best reliability and validity results among the model averaging techniques.

**Table 1:** Simulation results for the 1-aminoanthraquinone dataset using the benchmark dose method (reference model: Probit)

Exclusion criteria^**a**^	Selection^**b**^	Reliability (Mean distance)^**c**^	Rank	Validity^**d**^ (%)	Rank	BMDL calculability^**e**^ (%)	Non-exclusion and BMDL calculation^**f**^ (%)	True dose-response^**g**^ (%)
None	Lowest BMDL	0.4	5	100.0	1	95.6	95.6	0.1
	Lowest BMD	0.3	2	100.0	1	95.6	95.6	0.1
	Lowest AIC	120.9	15	88.4	15	95.6	95.6	34.1
	MA-all	6.2	9	99.6	8	95.6	95.6	NA
	MA-3	4.7	7	99.8	7	100.0	100.0	NA
	MA AIC < 3	9.0	11	98.8	11	100.0	100.0	NA
KS	Lowest BMDL	0.4	5	100.0	1	95.6	95.6	0.1
	Lowest BMD	0.3	2	100.0	1	95.6	95.6	0.1
	Lowest AIC	120.9	15	88.4	15	95.6	95.6	34.1
	MA-all	6.1	8	99.6	8	95.6	95.6	NA
KS, BMD/BMDL	Lowest BMDL	0.3	4	100.0	1	95.6	79.1	0.5
	Lowest BMD	0.2	1	100.0	1	95.6	79.1	0.5
	Lowest AIC	121.0	17	88.4	15	95.6	79.1	38.4
	MA-all	6.3	10	99.3	10	95.6	79.1	NA
KS, BMDU/BMDL	Lowest BMDL	27.2	13	91.0	12	95.6	49.0	18.4
	Lowest BMD	27.1	12	91.0	12	95.6	49.0	18.4
	Lowest AIC	148.3	18	79.4	18	95.6	49.0	35.3
	MA-all	33.9	14	90.2	14	95.6	49.0	NA

^a^Exclusion criteria: KS, Kolmogorov-Smirnov test of goodness-of-fit; BMD/BMDL, ratio of benchmark dose (BMD_10_) to benchmark dose lower bound (BMDL_10_) with values > 10 excluded; BMDU/BMDL, ratio of benchmark dose upper bound (BMDU_10_) to BMDL_10_ with values > 10 excluded. ^b^Model selection criteria: Lowest BMDL, model with the lowest value of BMDL_10_; Lowest BMD, model with the lowest value of BMD_10_; Lowest AIC, model with the lowest AIC value; MA-all, model averaging of all converged models; MA-3, model averaging of three models with the three lowest AIC values; MA-AIC, model averaging of all models with AIC values < 3 compared with the best model that yielded the minimum AIC. ^c^Reliability (Mean distance), measured as the mean distance between unbiased BMDL_10_ and calculated BMDL_10_ followed by rank. ^d^Validity (%), measured as the iterations that satisfied calculated BMDL_10_ lower than unbiased BMD_10_ followed by rank. ^e^BMDL calculability (%), measured as the iterations that yielded BMDL in the model selection criterion. ^f^Non-exclusion and BMDL calculation (%), measured as the iterations that yielded BMDL in the model selection criterion along with exclusion criteria. ^g^True dose response (%), measured by the default model selected by the model selection criterion. Note: Validity (%), BMDL calculability (%), non-exclusion and BMDL calculation (%), and true dose response (%) were converted into rates of iterations divided by 9000, nine models in 1000 simulation data. NA, not applicable

Similarly, simulation results for the 2-ethylhexyl vinyl ether dataset, which contained frequent testing at doses with low response rates, are shown in Table 2. BMDL_10_ was 24.69 and BMD_10_ was 28.65 under the selection of the Probit model as the unbiased model (Fig. 3). The validity was highest using the lowest BMDL or the lowest BMD for model selection whenever the same model exclusion was applied in advance. Model averaging, especially, MA-3 yielded the best reliability performance. Model exclusion did not improve the validity, rather it decreased the reliability estimates of MA-all. No model exclusion changed the calculability of BMDL, and only a small improvement in the reliability of MA-all was obtained by model exclusion.

**Table 2:** Simulation results for the 2-ethylhexyl vinyl ether dataset (reference model: Probit)

Exclusion^**a**^	Selection^**b**^	Reliability^**c**^ (Mean distance)	Rank	Validity^**d**^ (%)	Rank	BMDL calculability^**e**^ (%)	Non-exclusion and BMDL calculation^**f**^ (%)	True dose-response^**g**^ (%)
None	Lowest BMDL	17.8	7	100.0	1	85.2	85.2	0
	Lowest BMD	17.8	7	100.0	1	85.2	85.2	0
	Lowest AIC	20.7	15	66.7	9	85.2	85.2	33.4
	MA-all	6.2	6	66.7	9	85.2	85.2	NA
	MA-3	3.6	1	66.7	9	100.0	100.0	NA
	MA AIC < 3	5.1	2	66.7	9	100.0	100.0	NA
KS	Lowest BMDL	17.8	7	100.0	1	85.2	85.2	0
	Lowest BMD	17.8	7	100.0	1	85.2	85.2	0
	Lowest AIC	20.7	15	66.7	9	85.2	85.2	33.4
	MA-all	6.2	5	66.7	9	85.2	85.2	NA
KS, BMD/BMDL	Lowest BMDL	17.8	7	100.0	1	85.2	85.2	0
	Lowest BMD	17.8	7	100.0	1	85.2	85.2	0
	Lowest AIC	20.7	15	66.7	9	85.2	85.2	33.4
	MA-all	6.1	4	66.7	9	85.2	85.2	NA
KS, BMDU/BMDL	Lowest BMDL	17.8	7	100.0	1	85.2	85.2	0
	Lowest BMD	17.8	7	100.0	1	85.2	85.2	0
	Lowest AIC	20.7	15	66.7	9	85.2	85.2	0
	MA-all	6.1	3	66.7	9	85.2	85.2	33.4

^a^Exclusion criteria: KS, Kolmogorov-Smirnov test of goodness-of-fit; BMD/BMDL, ratio of benchmark dose (BMD_10_) to benchmark dose lower bound (BMDL_10_) with values > 10 excluded; BMDU/BMDL, ratio of benchmark dose upper bound (BMDU_10_) to BMDL_10_ with values > 10 excluded. ^b^Model selection criteria: Lowest BMDL, model with the lowest value of BMDL_10_; Lowest BMD, model with the lowest value of BMD_10_; Lowest AIC, e model with the lowest AIC value; MA-all, model averaging of all converged models; MA-3, model averaging of three models with the three lowest AIC values; MA-AIC, model averaging of all models with AIC values < 3 compared with the best model that yielded the minimum AIC. ^c^Reliability (Mean distance), measured by the mean distance between unbiased BMDL_10_ and calculated BMDL_10_ followed by rank. ^d^Validity (%), measured as the iterations that satisfied calculated BMDL_10_ lower than unbiased BMD_10_ followed by rank. ^e^BMDL calculability (%), measured as the iterations that yielded BMDL in the model selection criterion. ^f^Non-exclusion and BMDL calculation (%), measured as the iterations that yielded BMDL in the model selection criterion along with exclusion criteria. ^g^True dose response (%), measured by the default model selected by the model selection criterion. Note: Validity (%), BMDL calculability (%), non-exclusion and BMDL calculation (%), and true dose response (%) were converted into rates of iterations divided by 9000, nine models in 1000 simulation data. NA, not applicable

The simulation results for the acrylamide dataset, which contained doses involving both high and low response rates, are shown in Table 3. BMDL_10_ was 0.79 and BMD_10_ was 0.94 under the selection of the logistic model as the unbiased model (Fig. 3). The validity was highest using the lowest BMDL for model selection, and reliability was best when MA-3 was used. The logistic model, the unbiased dose-response curve for acrylamide, was selected for about every 1 in 3 selected models. The sample size in this dataset was larger than those in the other two datasets, the models converged at a higher frequency than they did in the other simulations and were rarely excluded by the BMD/BMDL or BMDU/BMDL ratio.

**Table 3:** Simulation results for the acrylamide dataset (reference model: Logistic)

Exclusion^**a**^	Selection^**b**^	Reliability^**c**^ (Mean distance)	Rank	Validity^**d**^ (%)	Rank	BMDL calculability^**e**^ (%)	Non-exclusion and BMDL calculation^**f**^ (%)	True dose-response^**g**^ (%)
None	Lowest BMDL	0.4	17	99.9	1	99.8	99.8	0.0
	Lowest BMD	0.3	13	99.7	5	99.8	99.8	0.1
	Lowest AIC	0.1	2	89.0	16	99.8	99.8	38.3
	MA-all	0.2	10	98.2	11	99.8	99.8	NA
	MA-3	0.1	1	93.9	14	100.0	100.0	NA
	MA AIC < 3	0.2	8	97.8	13	100.0	100.0	NA
KS	Lowest BMDL	0.4	17	99.9	1	99.8	99.8	0.0
	Lowest BMD	0.3	13	99.7	5	99.8	99.8	0.1
	Lowest AIC	0.1	2	89.0	15	99.8	99.8	38.3
	MA-all	0.2	9	98.4	10	99.8	99.8	NA
KS, BMD/BMDL	Lowest BMDL	0.3	16	99.9	1	99.8	98.7	0.0
	Lowest BMD	0.3	12	99.7	5	99.8	98.7	0.2
	Lowest AIC	0.1	5	88.7	17	99.8	98.7	38.7
	MA-all	0.2	7	98.7	9	99.8	98.7	NA
KS, BMDU/BMDL	Lowest BMDL	0.3	15	99.9	1	99.8	93.9	0.0
	Lowest BMD	0.2	11	99.6	8	99.8	93.9	0.2
	Lowest AIC	0.1	4	88.5	18	99.8	93.9	39.0
	MA-all	0.2	6	98.2	11	99.8	93.9	NA

^a^Exclusion criteria: KS, Kolmogorov-Smirnov test of goodness-of-fit; BMD/BMDL, ratio of benchmark dose (BMD_10_) to benchmark dose lower bound (BMDL10) with values > 10 excluded; BMDU/BMDL, ratio of benchmark dose upper bound (BMDU_10_) to BMDL_10_ with values > 10 excluded. ^b^Model selection criteria: Lowest BMDL, model with the lowest value of BMDL_10_; Lowest BMD, model with the lowest value of BMD_10_; Lowest AIC, model with the lowest AIC value; MA-all, model averaging of all converged models. MA-3, model averaging of three models with three smallest AIC values. MA-AIC, model averaging of all models with AIC values < 3 compared with the best model that yielded the minimum AIC. ^c^Reliability (Mean distance), measured as the mean distance between unbiased BMDL_10_ and calculated BMDL_10_ followed by rank. ^d^Validity (%), measured as the iterations that satisfied calculated BMDL_10_ lower than unbiased BMD_10_ followed by rank. ^e^BMDL calculability (%), measured as the iterations that yielded BMDL in the model selection criterion. ^f^Non-exclusion and BMDL calculation (%), measured as the iterations that yielded BMDL in the model selection criterion along with exclusion criteria. ^g^True dose response (%), measured as the default model selected by the model selection criterion. Note: Validity (%), BMDL calculability (%), non-exclusion and BMDL calculation (%), and true dose response (%) were converted into rates of iterations divided by 9000, nine models in 1000 simulation data. NA, not applicable

## Discussion

The BMD method is now widely used to determine the reference dose for toxicological risk assessment in food chemicals, agricultural chemicals, and environmental hazards. However, governmental experts are often puzzled by several ambiguous parts of model assessment, especially the model exclusion and selection processes. As part of the technical assessment for possible improvements in the guidelines, we conducted a simulation-based experiment to assess the model exclusion and selection process by comparing the validity, reliability, and other model performance indicators using all possible combinations of model exclusion and selection criteria. For the exposition, we examined three different empirical datasets, each with different characteristics of the dose-response pattern (i.e. the datasets had rich information about high or low response rates, and approximately linear dose-response patterns). By replicating 1000 sets of hypothetical experimental data computationally in a random manner, we found that the best criteria of model exclusion and selection were different across the chemical substances in each dataset. Further, the best criteria for achieving good validity was not necessarily the best for ensuring good reliability. For instance, the lowest BMDL outperformed the other criteria in achieving high validity, but did not always yield the best reliability. The use of lowest AIC yielded the best reliability result for the acrylamide dataset, but the worst reliability for the 1-aminoanthraquinone dataset. Besides, the model averaging results always ranked at an intermediate level among all possible criteria, and did not yield the worst results.

There are two take-home messages. First, although we did not identify the best exclusion and selection criteria for the qualitatively differently distributed datasets, we have shown that model averaging over three models with the lowest three AIC values (MA-3) did not yield the worst result, and MA-3 without prior model exclusion produced the best results among all the model averaging results. For instance, MA-3 yielded the best reliability result for the 2-ethylhexyl vinyl ether dataset. If a uniform guideline to implement model exclusion and selection is required, our results indicate that MA-3 could become the recommended option whenever applicable. Second, we found that model exclusion using the KS test and the ratios of BMD or BMDU to BMDL did not necessarily yield better validity and reliability than non-exclusion. In particular, both the validity and reliability for the 1-aminoanthraquinone dataset were made worse by imposing exclusion. For example, by applying the exclusion criteria of KS test and the ratio of BMDU and BMDL, reliability (mean distance) of Lowest BMDL has been increased from 0.4 to 27.2 as compared with non-exclusion (Table 1). In contrast, validity (rate of “successful” calculation) of MA-all has been decreased from 98.8 without exclusion to 90.2 as applied of KS test and the ratio of BMDU and BMDL (Table 1). Thus, to decide about model exclusion, visual assessment might be enough to proceed to model selection.

Model averaging has previously been demonstrated as a useful option when determining the point of departure [25], especially for datasets that do not necessarily exhibit a sigmoidal dose-response curve. We found that all the model averaging options that we tested performed well overall. However, how the distance metric (e.g. AIC) across different models can be account for and how model uncertainty of each parametric assumption in the process of averaging can be quantified still need to be considered. Considering that at least nine models are fitted to the same dataset and some of the models share similar properties while others do not, which models should be averaged needs to be considered, e.g. averaging over all models or only some of them. We found that averaging over some of the models might yield a better performance than averaging over all converged models, considering that the uncertainties of well-fitted models might be far smaller than those of badly fitted models. Averaging over the three best models is still a subject of debate (e.g., averaging over two best models rather than three) and the numbers might change depending on the total number of models to be tested (e.g. more than nine models could be tested) [25]. However, considering that averaging over the three best models outperformed all the models with close AIC values, reliance only on the penalized likelihood during the averaging might not be a good option. For now, MA-3 is the method that we recommend, and we plan to share the programing code and a package for this procedure in the future.

It must be noted that the recommended option does not work when the total number of converged models is one or two; indeed, the convergence of one model alone can occur occasionally. In such an instance, other criteria, including using the modeling results that yield the lowest BMDL or the model with the lowest AIC value, need to be considered. What the present study has shown is that both the lowest BMDL and lowest AIC did not act as the unique best method for model selection, whereas the lowest BMDL method can ensure good validity, which is understandable from the conservative nature of this method. It should be noted that the use of the lowest AIC was ranked as part of the worst result for two of the datasets (the exception was the acrylamide dataset) when it comes to validity and reliability.

Five technical limitations should be considered. First, we examined only three different chemical substances as source of information and addressed qualitative differences only among the three datasets. More datasets may have revealed additional insights into ranking the model selection criteria. Second, if a specific dataset behaved uniquely, there should be a corresponding unique criterion that is best suited to its analysis. However, our objective was to identify acceptable model selection criteria across qualitatively different datasets (which found MA-3 was acceptable overall) and we were not able to classify dose-response curves into several different groups for better fitting. Third, we used only computer simulations. Using the reference model prior to simulations might have been preferred during the estimation process. Although we counted this bias in Tables 1, 2 and 3, the impact of this on our examined criteria is not known. Fourth, we did not explore parameter constraints in this study. Fifth, we did not examine other percentile cutoff levels, i.e. the benchmark response was fixed at 10%.

While numerous technical issues have yet to be explored in applying BMD methods to risk assessment, we concluded that MA-3 can be considered the best guiding option to derive the reference dose when the guidelines are expected to specify a single model exclusion and selection method.

## Conclusion

As part of the technical assessment for possible improvements in the guidelines, we conducted a simulation-based experiment to assess the model exclusion and selection process by comparing the validity, reliability, and other model performance indicators using all possible combinations of model exclusion and selection criteria. If a uniform methodological suggestion for the guideline is required to choose the best performing model for exclusion and selection, our results indicate that using MA-3 is the recommended option whenever applicable.

## Data Availability

The datasets used in this study are publicly available and can be retrieved from [26–28]. The simulated data generated during this study will be shared electronically by the corresponding author upon reasonable request.

## Abbreviations

AIC: Akaike Information Criteria
BMD: Benchmark dose
BMDL: Benchmark dose lower bound
BMDU: Benchmark dose upper bound
CI: Confidence interval
MA: Model averaging
MA-3: Model averaging over three models with the lowest three AIC
MA AIC < 3: Model averaging over models with the difference of AIC less than 3 from the best-fit model with the lowest AIC value
MA-all: Model averaging over all converged models
NOAEL: No observable adverse effect level
